# Effect of VERO pan‐tilt motion on the dose distribution

**DOI:** 10.1002/acm2.12112

**Published:** 2017-06-06

**Authors:** Heru Prasetio, Indra Yohannes, Christoph Bert

**Affiliations:** ^1^ Department of Radiation Oncology Universitätsklinikum Erlangen Friedrich‐Alexander‐Universität Erlangen‐Nürnberg Erlangen Germany

**Keywords:** Dose distribution, dosimetry, gamma evaluation, gimbal tracking

## Abstract

Tumor tracking is an option for intra‐fractional motion management in radiotherapy. The VERO gimbal tracking system creates a unique beam geometry and understanding the effect of the gimbal motion in terms of dose distribution is important to assess the dose deviation from the reference conditions. Beam profiles, output factors (OF) and percentage depth doses (PDD) were measured and evaluated to investigate this effect. In order to find regions affected by the pan‐tilt motion, synthesized 2D dose distributions were generated. An evaluation of the 2D dose distribution with the reference position was done using dose difference criteria 1%–4%. The OF and point dose at central axis were measured and compared with the reference position. Furthermore, the PDDs were measured using a special monitoring approach to filtering inaccurate points during the acquisition. Beam profiles evaluation showed that the effect of pan‐tilt at inline direction was stronger than at the crossline direction. The maximum average deviation of the full width half maximum (FWHM), flatness, symmetry, penumbra left and right were 0.39 ± 0.25 mm, 0.62 ± 0.50%, 0.76 ± 0.59%, 0.22 ± 0.16 mm, and 0.19 ± 0.15 mm respectively. The ÔF and the measured dose average deviation were <0.5%. The mechanical accuracies during the PDD measurements were 0.28 ± 0.09 mm and 0.21 ± 0.09 mm for pan *and* tilt and pan *or* tilt position. The PDD average deviations were 0.58 ± 0.26 % and 0.54 ± 0.25 % for pan‐*or‐*tilt and pan‐*and‐*tilt position respectively. All the results showed that the deviation at pan *and* tilt position are higher than pan *or* tilt. The most influences were observed for the penumbra region and the shift of radiation beam path.

## 1 INTRODUCTION

Motion management plays an important role in an advanced external beam radiotherapy treatment, especially when the target and organ at risk (OAR) are moving during the treatment delivery. Mitsubishi (Heavy Industry, Ltd., Japan) and Brainlab (Feldkirchen, Germany) have developed a linac system called VERO that is capable of compensating intra‐fractional motion.^1^ The machine is coupled with a dedicated Exactrac VERO infra‐red stereo camera and a dual tube fluoroscopy tracking system. Those systems continuously monitor the patient and estimate the target motion coordinate during treatment.^1^, ^2^

The predicted target coordinates are passed to the gimbal head controller of the accelerator, which adapts accordingly to compensate the target motion. Eventually, the reduced intra‐fractional uncertainties will potentially shrink CTV‐PTV margins.^3^ In comparison to other motion mitigation strategies, the treatment time can potentially be reduced since the beam is delivered continuously while the target is in motion.^1^, ^2^, ^3^, ^4^, ^5^, ^6^, ^7^, ^8^, ^9^, ^10^

Instead of moving its gantry or multi leaf collimator (MLC) leaves for tracking, VERO swings the gimbal head. It has its own center of rotation, which is located at 40 mm below the source.^1^, ^2^ Therefore, the geometry of the beam during tracking is different from a common oblique beam, which is created by moving the linac gantry. The gimbal rotation is currently not supported by available treatment planning systems (TPS)^3^, ^4^, ^5^, ^6^, ^7^, ^8^ and thus potentially leads to an inaccurate treatment delivery. Such systematic errors will not be noticeable during the dose calculation. Currently, the gimbal tracking dose calculation is performed on a stationary CT without considering the gimbal's motion. Dose calculation is thus relying only on shifting of the target into the radiation field,^3^ which is not giving a similar radiation path of the beam. Some dosimetry studies showed that the gimbal motions will not affect the beam profile characteristics, i.e., beam profile and penumbra agreed within 1%/1 mm.^5^, ^6^ Furthermore, deviations in output factor (OF), percentage depth dose (PDD) and 2D dose distribution were not observed.^5^ All the previous studies used films as their primary detectors.^5^, ^6^

The aim of this work was to investigate the effect of pan‐tilt motions during treatment on the delivered dose distribution in a water phantom. However, the situation is more complex in the real clinical condition, since the patient surface, tissue density, and tumor motion direction will greatly influence the dose calculation and the treatment accuracy during the treatment. A comprehensive evaluation regarding the effect of the VERO's gimbal motion on fundamental dosimetry properties such as beam profile, OF, and PDD was performed to provide a better understanding of the gimbal motion effects on the delivered dose. Furthermore, it will help medical physicists to estimate the accuracy of treatment delivery.^11^, ^12^, ^13^, ^14^, ^15^ Especially, if the tracking treatment is combined with other complex treatments such as intensity modulated radiotherapy (IMRT), or wavearc^16^ treatments.

## 2 MATERIALS AND METHODS

### 2.A Measurement geometry and setup

**Figure 1:**
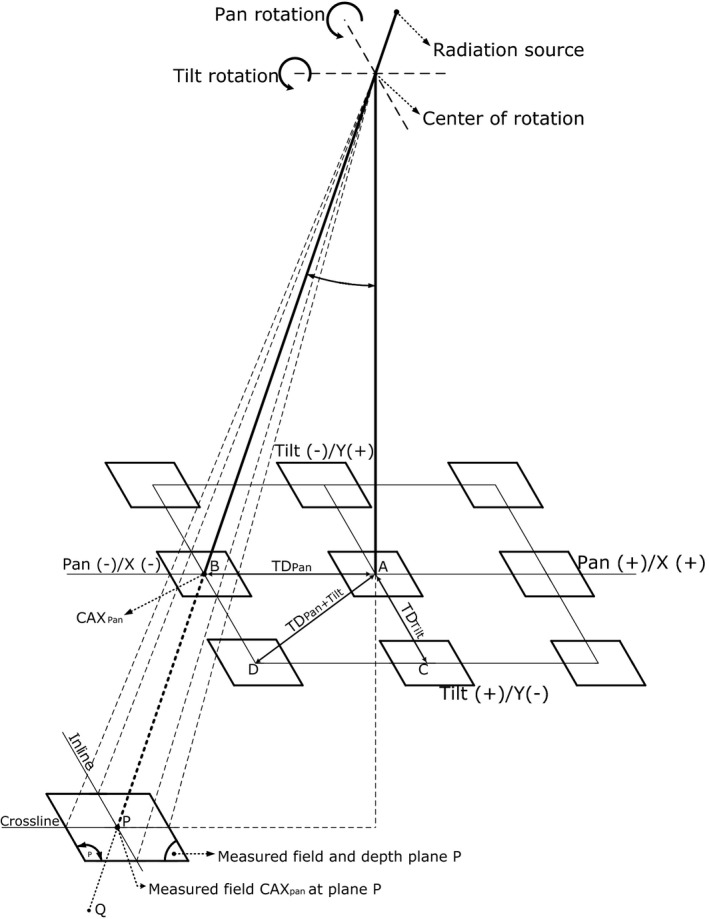
Schematic diagram of the gimbal motion geometry during pan/tilt position. Points A, B, and P are the machine central axis at the water surface, and measured plane during the gimbal motion at pan position respectively. Comparable to a pan position (point B) is the gimbal at a tilt (point C) or gimbal at pan *and* tilt (point D). The gimbal center of rotation is located 40 mm below the radiation source, which creates an SSD 960 mm without pan *or* tilt.

**Figure 2:**
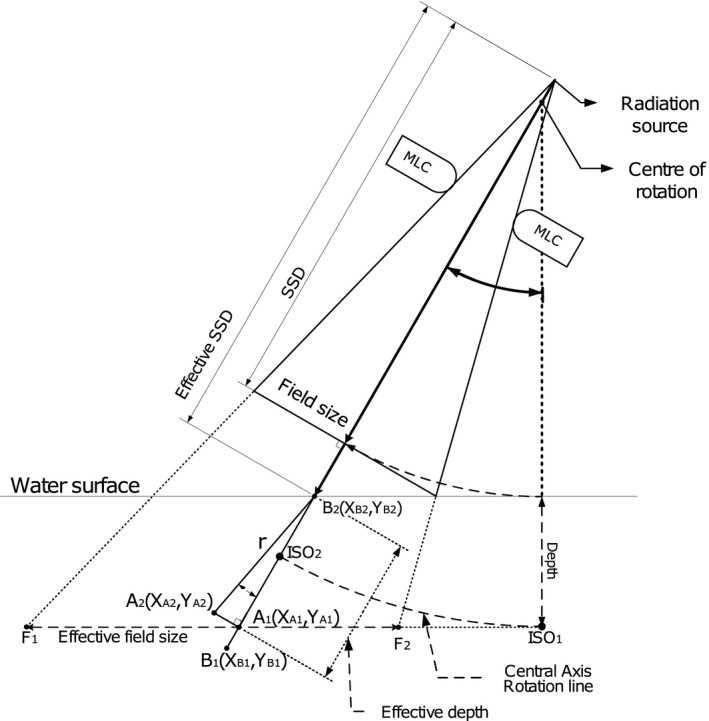
Beam geometry properties for the beam profile, PDD and OF measurements at pan‐tilt position, with α being the pan‐tilt angle according to the tracking distance (TD). The beam profiles, PDDs and OFs were measured from point F_1_ to F_2_, B_1_ to B_2_, and at A_1_ respectively. The isocenter(ISO_1_ and ISO_2_) is separated with the target plane during tracking, due to the fix distance of the isocenter.

As shown in Fig. 1, the VERO gimbal can be swung to inline/tilt (A→C) and crossline/pan (A→B) direction using its center of rotation (COR). A target tracking utilizing the gimbal motion creates a unique beam geometry as shown in Fig. 2. Unlike the common oblique beam geometry, the gimbal geometry creates a longer source surface distance (SSD) and a larger effective field size (FS). The gimbal can track a moving target up to a maximum tracking distance $\left(TD\right)\;=\;41.9\;\mathrm{mm}$ away from the isocenter at a pan *or* tilt directions, which is equal to an angle (α) of pan or tilt of 2.5 degree (Fig. 1). Moreover, a maximum gimbal position at a pan *and* tilt position (A→D) will result in a relative angle of the gimbal to the water surface of 3.5 degree (Fig. 1). This creates an SSD increase of 1.83 mm compared to the reference. Since beam profile, OF and PDD are influenced by the FS and SSD,^17^, ^18^ both parameter changes could leads to a deviation of the reference dosimetry parameters.

All measurements in this study were carried out using BLUE PHANTOM^2^ (IBA Dosimetry GmbH, Schwarzenbruck, Germany) and a microDiamond single crystal detector (T60019, PTW‐Freiburg GmbH, Freiburg, Germany). The reference condition was defined at pan and tilt angle 0^o^. Moreover, the PDDs and profile measurements were done using an output resolution of 1 mm and the field detector reading was normalized at a depth of 15 mm.

The central axis (CAX) position played an important role during the measurement since it was used as a reference to determine the scanning region. Moreover, it ensured that the scanning profile was always intersected at the CAX position (Figs. 1 and 2). Therefore, the PDD scan direction, as well as the OF depth, were always along the CAX beam path. The coordinates of the CAX at a certain depth (d) were determined by calculating the tracking angle (α) based on the TD of the gimbal at pan‐tilt angle (Fig. 1) using the COR_SSD_ at the reference position, which was 960 mm:

(1) $$\alpha_{\mathrm{pan}/\mathrm{tilt}}=\mathrm{ta}n^{-1}\left(\frac{TD_{\mathrm{pan}/\mathrm{tilt}}}{COR_{SSD}}\right)$$

(2) $$\mathrm{CA}X_{\mathrm{pan}/\mathrm{tilt}}=\tan\left(\alpha_{\mathrm{pan}/\mathrm{tilt}}\right)\times \left(COR_{\mathrm{SSD}}+\;d\right)$$

### 2.B Beam profile and output factor

Beam profiles were measured as described in Table 1, i.e., in total of 720 profiles. The scanning regions were always set to 300 mm at inline and crossline directions for all FSs and depths. The start (F_1_) and stop (F_2_) position at inline and crossline directions were set 150 mm away from CAX in both directions as shown if Fig. 2. Profiles were measured parallel to the water surface, i.e., involving only one of the motors of the phantom for each measurement. Output factor measurements were performed as described in Table 1, i.e., 360 OFs were measured. The detector inline (tilt) and crossline (pan) coordinate at each corresponding depth position (d) were calculated using Eq. (2).

**Table 1:** Summary of measurement combinations and parameters

Pan distance (mm)	:	−41.9, 0, 41.9
Tilt distance (mm)	:	−41.9, 0, 41.9
Squared field size (mm)	:	10, 20, 30, 40, 50, 60, 80, 100, 120, 150
Depth at CAX (mm)	:	15, 50, 100, 200
Measured parameters	:	Beam profiles at inline and crossline PDD Output factor (OF)

### 2.C Percentage depth dose

A similar approach as the OF measurements was applied to determine the start‐stop position for the PDD measurements. The start and stop positions on the CAX were calculated using Eqs. (1) and (2) at a depth of 300 mm and 0 mm respectively. The 3D dose scan mode was used for the PDD measurement.

A dry run test was performed prior the measurement to ensure a detector position at CAX of the beam. The test also functioned as detector positioning consistency and mechanical movement quality control of the BLUE PHANTOM^2^ during the 3D dose scan mode. After the initial dry run test, it was found that the mechanical movement of the detector arms was not always smooth, which could create a positioning inaccuracy. This unsmoothed movement might occur due to the movement of more than one motor in order to position the detector to the correct measurement position. Therefore, a protocol to monitor the detector arm positioning was implemented to minimize the mean positioning inaccuracy. The monitoring was done by calculating the distance (r) of the measured data point at $B_{1}$ to a designated scanning line of $\vec{B_{1}B_{2}}$, which was acting as expected path of measurement and point $B_{2}$ being equivalent with CAX at pan/tilt (Fig. 2). The closest distance (r) of each measurement point to the scanning line only occurred at a perpendicular position (Fig. 2). Additionally, r was calculated by projecting the measurement directional vector $\vec{A_{2}B_{2}}$ into the scanning line directional vector $\vec{B_{1}B_{2}}$. The distance of rwas determined by dividing the crossproduct of both vectors with the magnitude of the scanning vector $\vec{B_{1}B_{2}}$:

(3) $$r\left(\text{mm}\right)=\frac{\left|\right|\vec{A_{2}B_{2}}\times \vec{B_{1}B_{2}}\left|\right|}{\left|\right|\vec{B_{1}B_{2}}\left|\right|}$$

The effective measurement depth ($d_{\mathrm{eff}.}$) was determined by calculating its relative distance to the CAX coordinate:

(4) $$d_{\mathrm{eff}.}\left(\mathrm{mm}\right)=\sqrt{{\left(X_{A2}-X_{B2}\right)}^{2}+{\left(Y_{A2}-Y_{B2}\right)}^{2}+{\left(d\right)}^{2}}$$

Measurement point coordinate at inline (Y), crossline $\left(X\right)$ direction and depth $\left(d\right)$ were obtained from the measurement software output.

### 2.D 2D dose distribution synthesis

Additional evaluations were performed to understand the dosimetric effect of gimbal pan‐tilt motions. Using the profile data from inline and crossline measurement, the 2D dose distributions were generated by multiplication of the inline measurement along the crossline profile and normalized at CAX. The modeling of VERO in the Pinnacle^3^ treatment planning system (Philips Healthcare) has been implemented^19^ and the system was used to validate the synthesized field at 100 × 100 mm^2^ field size and a depth of 15, 50, 100, and 200 mm. Further evaluation and analysis, such as dose deviation evaluation, could be performed using this profile.

### 2.E Data analysis

Data evaluation and analysis were completed by comparing all pan‐tilt measurements with the respective reference defined at SSD = 100 cm and pan‐tilt 0^o^. The average deviation (Δ) of the measured pan‐tilt beam profile parameters with the reference was taken for an evaluation. The beam profile parameters analysis was conducted by evaluating the Δ of Full‐Width Half Maximum (FWHM), symmetry, flatness, and penumbra. The output factor comparison was made by calculating the OF relative average deviation with the reference.

The PDD assessments were obtained by comparing the dose difference at each depth according to Table 1 to the reference condition. The comparison was performed at depths beyond 15 mm or the build‐up region to avoid large uncertainties of the depth dose distribution close to the water surface due to electron scattering created by the beam‐shaping aperture above the water surface.^20^, ^21^

The calculated 2D dose distributions from inline and crossline measurements were assessed pixel‐by‐pixel. The approach was performed to study the effect of pan‐tilt movements on the dose distribution profile and to identify the region that is mostly influenced by the movement. The assessment was conducted using full field measurements using a 2% threshold as the limit of the radiation field. Secondly, the evaluation was done using 80% of the physical field size at the corresponding depth of measurement, which represents the effective field size for treatment.

## 3 RESULTS

Measurement data sets for a single pan‐tilt position consist of 10 field sizes, 4 depths, inline and crossline scan directions. Total measured data consist of 80 profiles with additional 10 PDD curves and 40 points of OF measurement for each position. In total there were 9 gimbal positions involved as shown in Fig. 1, which made a total data set of 720 profiles, 90 PDD curves and 360 points for the OF. Making use of inline and crossline profiles, a total number of 40 2D dose distributions were generated for each pan‐tilt position. Each data point for the OF measurement consists of minimum three repetitions to ensure the beam output consistency.

### 3.A Profile characteristics and output factor

**Figure 3:**
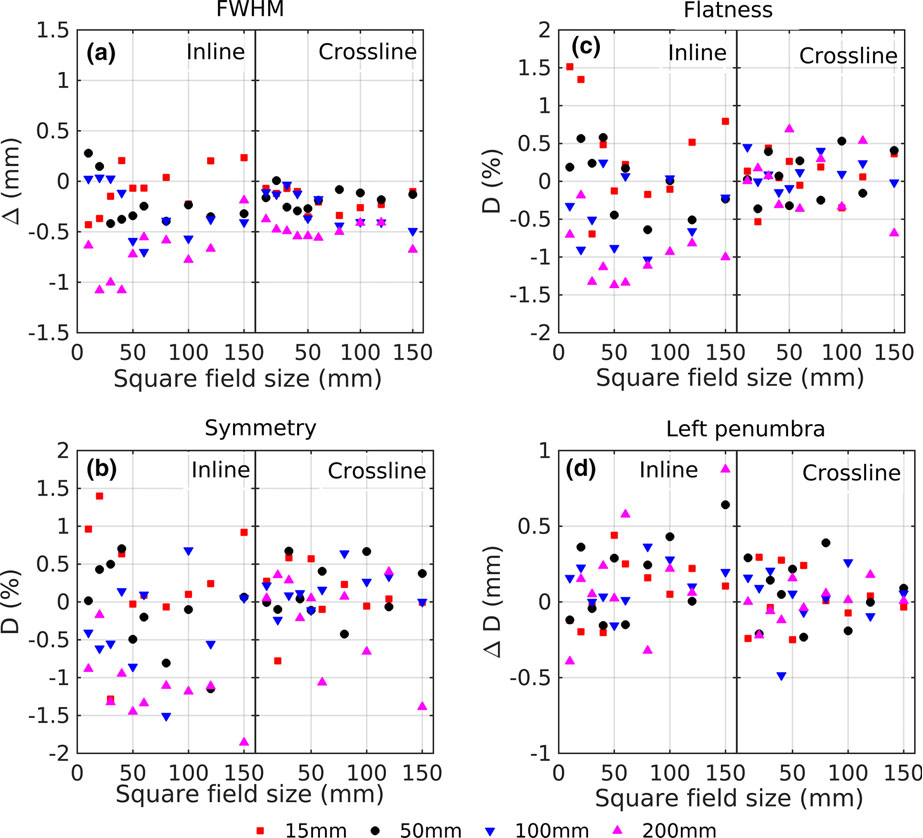
Deviation of FWHM (a), symmetry (b), flatness (c), and penumbra left (d) for inline and crossline direction at pan −41.9 mm and tilt 41.9 mm.

Calculation of beam profile characteristics, i.e., FWHM, flatness, symmetry, and penumbra were calculated and compared with the reference condition. Figure 3 is an example of the Δ at pan‐tilt positions −41.9 mm and 41.9 mm. The penumbra left and right shows similar patterns and values, therefore only the left penumbra is shown in Fig. 3.

Similar results were found at other pan‐tilt position as seen in Fig. 3. Since there was no clear pattern of the Δ, the evaluation of the beam profile characteristics related to pan‐tilt position, field size, and depths, further analysis was done by averaging the deviation values for all field sizes and depth at each pan‐tilt position. Generally, the effect of pan‐tilt position at inline direction was stronger than at crossline direction as shown in Fig. 3. All the maximum deviations occurred at inline profiles, which were 0.39 ± 0.25 mm, 0.62 ± 0.50%, 0.76 ± 0.59%, 0.22 ± 0.16 mm, and 0.19 ± 0.15 mm for FWHM, flatness, symmetry, penumbra left and right respectively.

**Figure 4:**
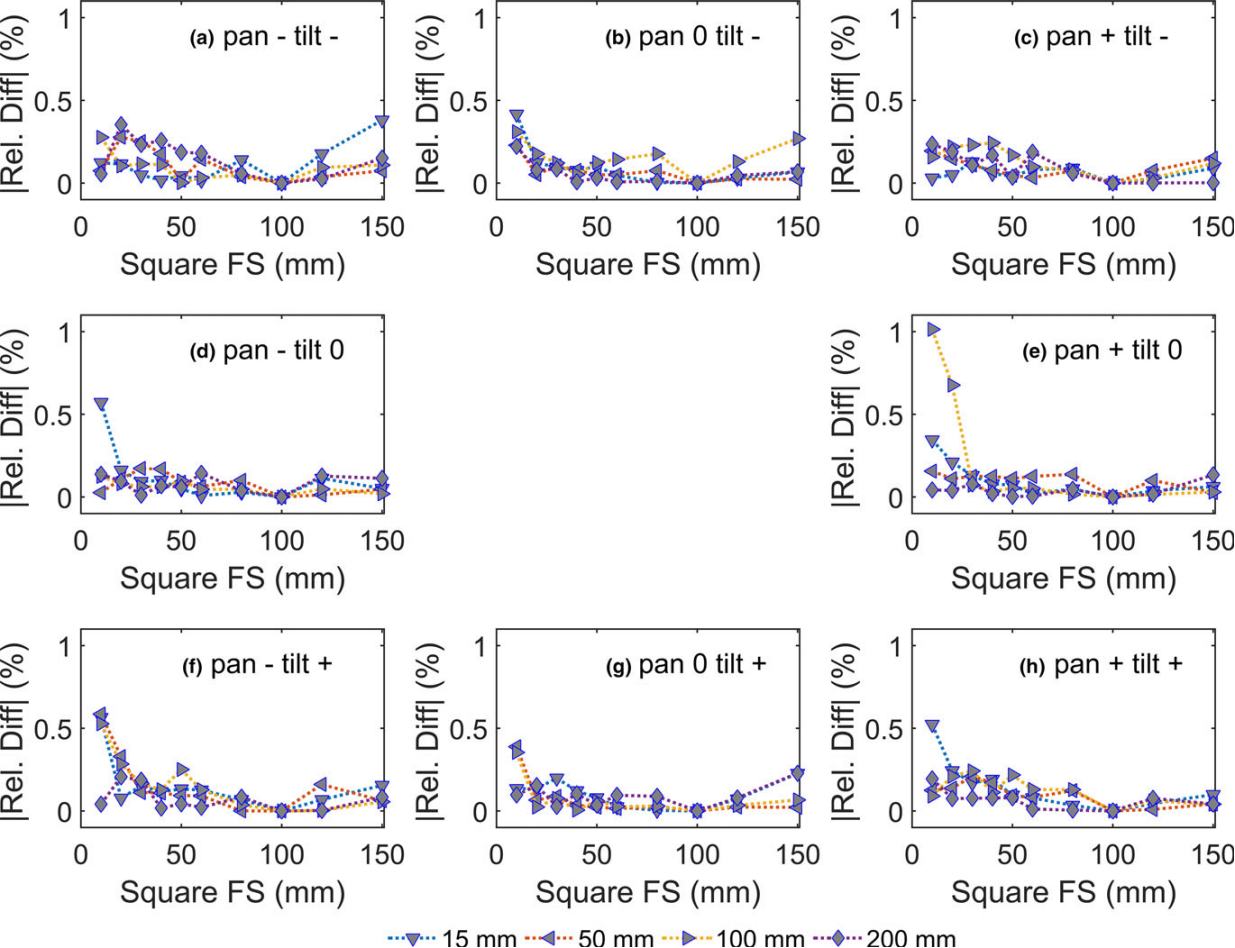
The relative differences of the output factor at all pan‐tilt positions with the reference. The overall layout of the figure corresponds to observer beam's eye view from the radiation beam direction.

**Figure 5:**
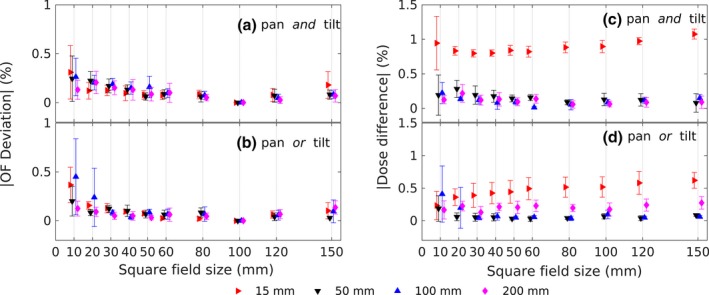
The OF deviation at pan *and* tilt (a) and pan *or* tilt (b) positions, and the dose difference from the detector reading during the output factor measurement (c and d). To ease the readability, the data points were shifted at each field size entry.

The OF relative difference pattern was similar for most of the field sizes as shown in Fig. 4. The summary of the OF deviation and the dose difference during the OF measurements are shown in Fig. 5. The mean difference values for pan *and* tilt and pan *or* tilt were less than 0.1%. The OF deviation shows a decreasing trend from 10 mm × 10 mm to 100 mm × 100 mm field size and then an increasing trend toward 150 mm × 150 mm. The dose measured during the OF measurements during pan‐tilt were lower than at the reference position. The average deviation of the measured dose at pan and tilt is slightly higher than at pan or tilt position, which was less than 0.1%.

### 3.B Mechanical movement monitoring

Unlike the profile measurements, the PDD measurements were more complex with respect to phantom mechanical movements since the detector positioning involved more than one motor. The measurement involving one motor movement did not show any positioning deviation since vector $\vec{A_{2}B_{2}}$ and $\vec{B_{1}B_{2}}$ will always overlap with a resulting deviation of r=0 (Fig. 2). Moreover, the other two motors will lock itself, as a result the remaining motor movement will always be on its vector direction.

**Figure 6:**
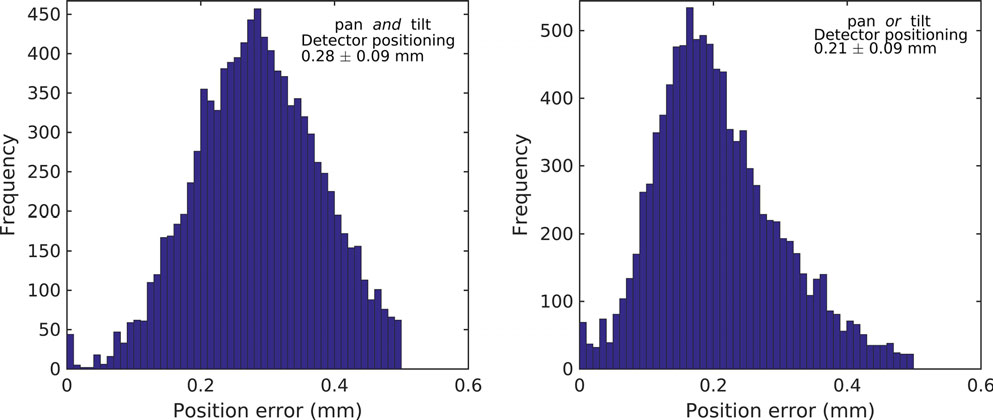
The mean detector positioning accuracy histogram after filtering detector positions with *r *> 0.5 mm during the PDDs measurement. Left: for pan *and* tilt motion, right for pan *or* tilt motion.

The dry run test showed unsmoothed mechanical movement since the motion was based on more than one motor. Maintaining the distance *r* of the measured point to the nominal scanning line less than 0.5 mm resulted in a mean positioning accuracy of 0.21 ± 0.09 mm and 0.28 ± 0.09 mm for 2 and 3 axes movements during the PDD measurement (Fig. 6), respectively.

### 3.C Percentage depth dose

**Figure 7:**
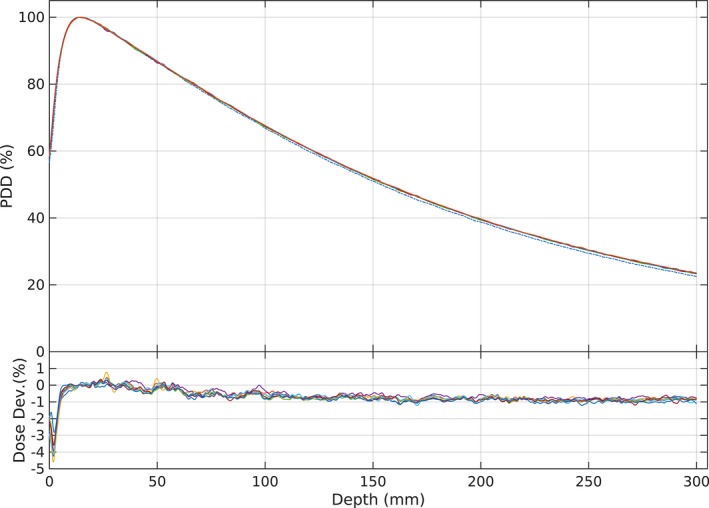
PDDs distributions at 100 mm × 100 mm field size at all pan‐tilt positions (―) and in reference condition (‐‐‐). The lower panel shows the dose difference of all PDDs with the reference field (―). Due to the PDDs and the differences.

**Figure 8:**
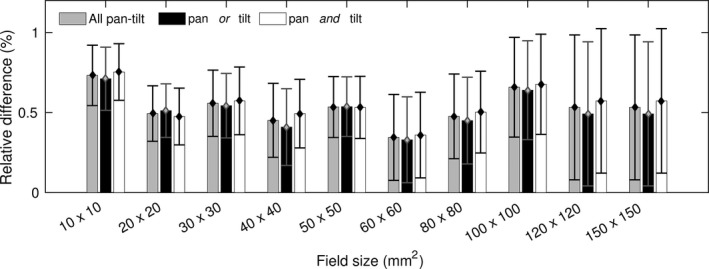
The PDD dose average deviation from its reference condition at all pan‐tilt positions for each field size.

Figure 7 shows an example of the filtered and corrected^21^ PDD measurements at 100 mm × 100 mm field size based on Eqs. (3) and (4). Irradiation at pan‐tilt position leads to higher PDD curves than the reference beyond the buildup region and lower values within the buildup region. In the analysis of all data, the dose values within the buildup region were omitted to reduce a bias on the mean dose deviation. The dose average deviation while gimbal at pan *and* tilt position was higher than at pan *or* tilt, as shown Fig. 8. The PDD average deviations at all field sizes at pan‐tilt position were less than 1% (Fig. 8). Figure 8 also shows that the average deviations at pan *and* tilt are higher than at pan *or* tilt position, since the SSD is longer than the pan *or* tilt position.

### 3.D The 2D dose distribution

**Figure 9:**
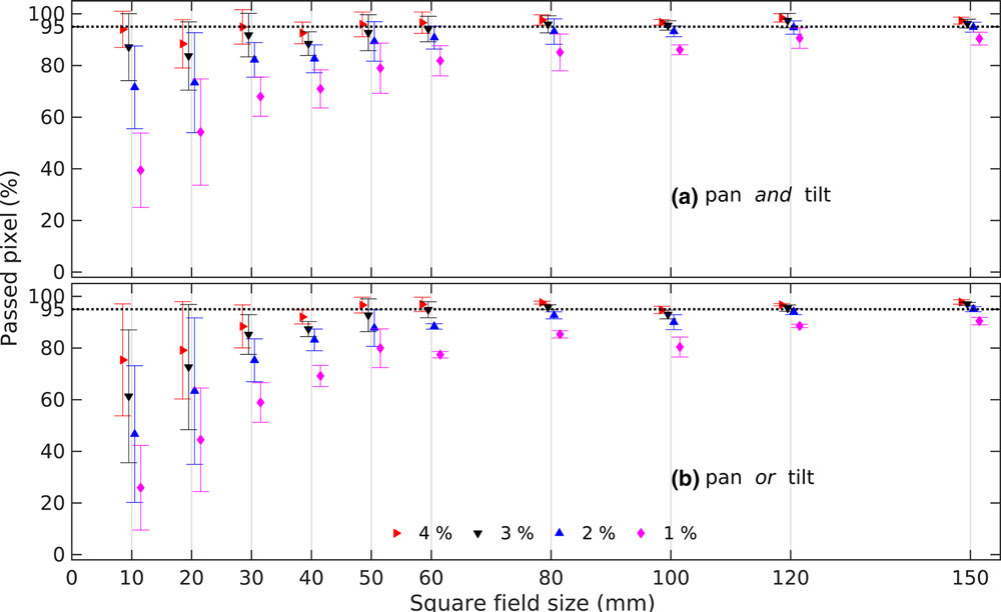
The percentage of passed pixels at depth 50 mm using 1%–4% dose difference criteria for pan *and* tilt (a) and pan or tilt (b). To ease readability the data points were shifted at each field size entry.

The synthesized dose distribution resulting from multiplying an inline and crossline dose profile at a corresponding depth has been compared with the calculated dose profile from the treatment planning system. The comparisons showed the smallest and largest mean difference between the synthesized and calculated dose profile of −0.1 ± 1.9% and 0.7 ± 0.7% respectively. The synthesized 2D dose distributions comparisons with the reference were evaluated using a pixel‐to‐pixel comparison and a criteria 1%–4% dose deviation was used to evaluate the dose distribution. Figure 9 shows that most of the profiles had less than 95% of the point that passed the criteria.

**Figure 10:**
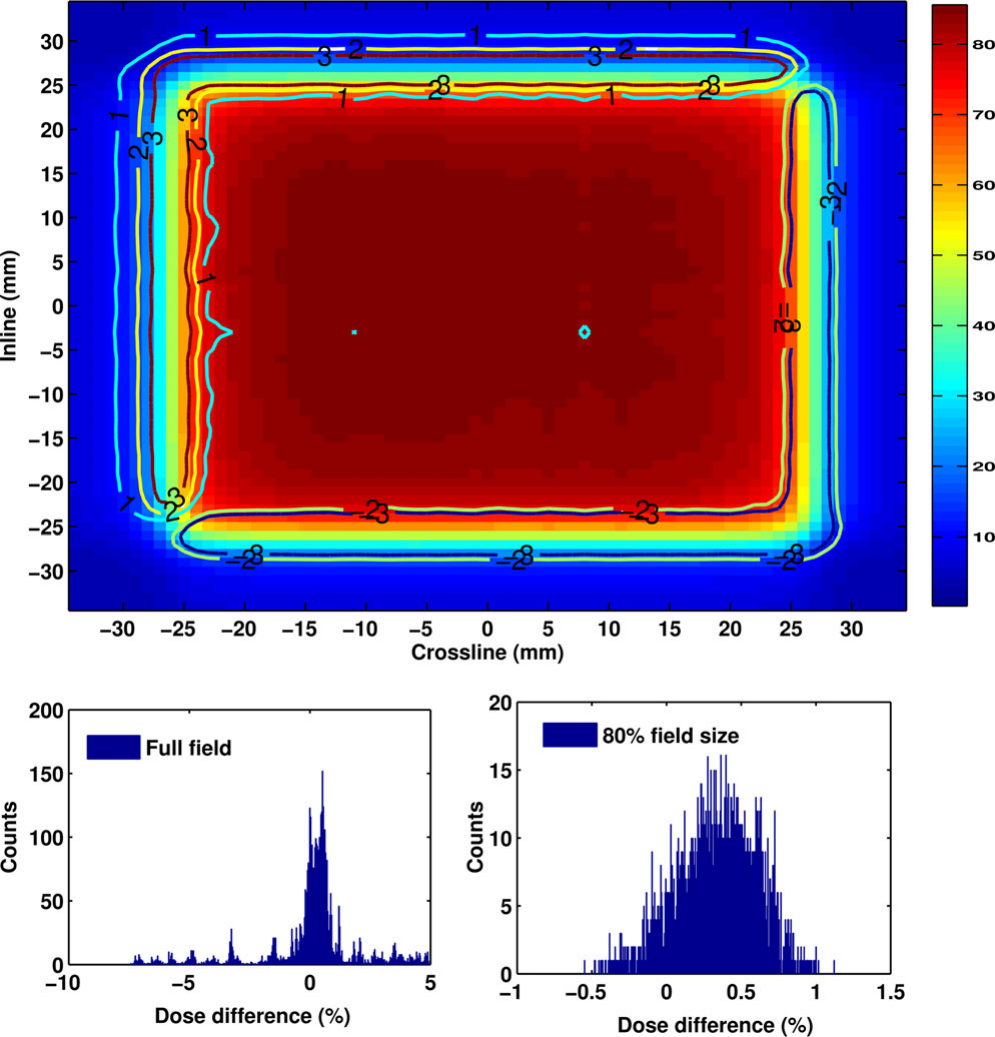
A 2D dose distribution and the corresponding histogram of a 2D dose pixel‐to‐pixel comparison at 50 mm × 50 mm field size, depth of 50 mm, pan‐tilt position of −41.9 mm and 41.9 mm. Overlaying the dose deviation contour with the dose profile showed that the penumbra is greatly affected by pan‐tilt motion. The color bar indicates the normalized dose distribution in % and the isodose line indicates a dose difference of the pan‐tilt position and the reference.

The pixel‐by‐pixel comparisons showed that the mean dose deviation was less than 1% with high dose deviations at the penumbra regions (Fig. 10), resulting in high standard deviations of its mean dose difference. Removing the penumbra region by comparing only 80% of the full field size for further evaluation showed that the mean dose deviations were similar, but the standard deviation was lower. The 80% region is an indication of the penumbra starting point that indicated as a starting of high dose gradient region. Avoiding a high dose gradient region would smooth the dose difference fluctuation. Overlapping the dose deviation distribution and the 2D dose distribution revealed the local deviation position (Fig. 10). The histogram of the full field comparison showed high dose deviations in the penumbra region and lower dose deviations were obtained at the comparison of the 80% full field size. Those values had strengthened the fact that high dose deviations occurred in the penumbra region. The average deviation of the 2D dose comparison for pan *and* tilt were higher than pan *or* tilt as shown in Table 2, and the values show a decreasing trend as the depth increases.

**Table 2:** The average deviation of 2D dose comparison based on 80% of full field size

Depth (mm)	Pan *and* tilt mean deviation (%)	Pan *or* tilt mean deviation (%)
15	0.49 ± 0.46	0.43 ± 0.39
50	0.49 ± 0.41	0.40 ± 0.35
100	0.40 ± 0.33	0.34 ± 0.27
200	0.28 ± 0.22	0.25 ± 0.20

### 3.E The radiation path

**Figure 11:**
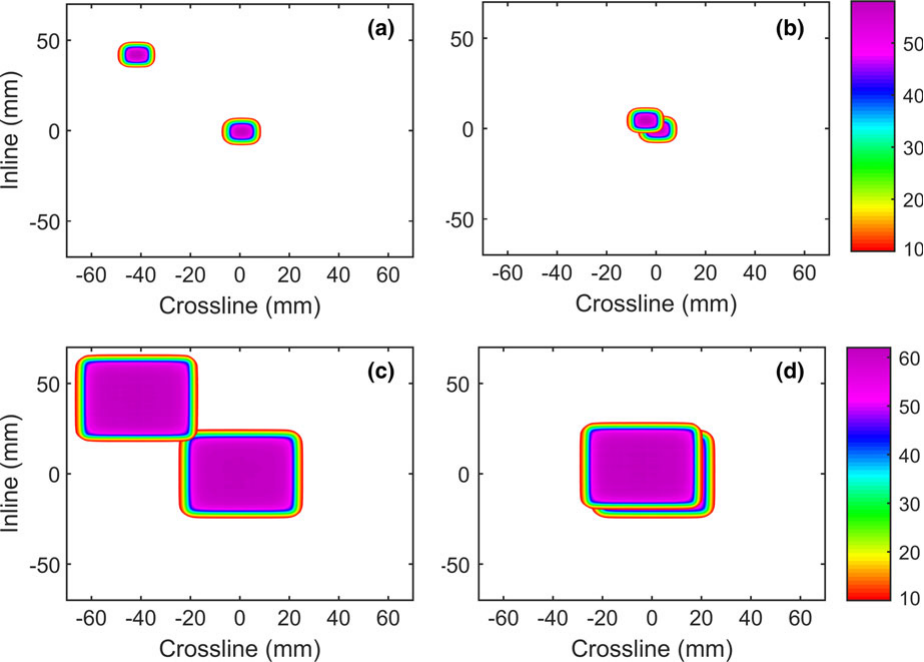
The condition of the pan and tilt irradiation at a maximum position for field sizes 10 mm × 10 mm (a) and 40 mm × 40 mm (c) at a depth of 50 mm away from the target position. Shifting the beam following the target position alone will result in miss irradiation to the region below the target (b and d).

The radiation paths of pan‐tilt are shown in Fig. 11. The position of the dose distribution is shifted according to the pan‐tilt position which depends on the pan‐tilt angle as described in Eqs. (1) and (2). The radiation path at CAX is shifted compare with its reference positions, as well as the dose distribution above and below the SAD point [Figs. 11(b) and 11(d)]. The effect of the radiation path shift is stronger as the field sizes getting smaller and the dose plane location is far from the target plane [Fig. 11(b)].

## 4 DISCUSSION

In this study, a comprehensive investigation regarding the effect of gimbal movements and its impact on the dose distributions has been conducted. The dose distribution parameters being observed were 2D dose distribution, OF, and PDD. Observation of the dose distribution deviation could help physicists to understand the effect of gimbal movements on the delivered dose.

We measured the dose distribution at maximum pan‐tilt position according to Table 1. However, there were no sufficient data sets to evaluate the effect of pan‐tilt movement toward pan and tilt angle 0^o^ at several small increments. Ideally, at least 5 points are required to create a proper statistic trend of the pan‐tilt effect compared to the reference, which is equal to 0.5^o^ angle increment. Reproducing of such a small angle motion during measurement was difficult to achieve while measuring using a BLUE PHANTOM.^2^ Additionally, a pinpoint detector with a small physical volume should ideally be used to measure the small lateral distance separation of each small angle increment, especially in the region closer the depth of maximum dose. Since such a detector was not available, it was not possible to evaluate the pan‐tilt effect based on a small pan‐tilt positioning increment. As a result, the evaluation only considered pan‐tilt position as one group data and compared directly to the reference condition. The deviation comparisons were done while gimbal is at the pan‐*and‐*tilt position, and pan‐*or*‐tilt position.

Beam profile comparison showed that the effect of pan‐tilt movement at inline directions was stronger than in crossline direction (Fig. 3). Kamino et al.^2^ described that the electron profile exiting from the accelerator waveguide in inline direction (Y) has a wider FWHM than in crossline direction (X). This situation creates a larger source size at Y direction, which eventually influences the flatness, symmetry and penumbra.

This study showed that the maximum deviation of FWHM, flatness, symmetry, and penumbra from the reference were (0.38 ± 0.27), (0.62 ± 0.50)%, (0.76 ± 0.59)%, and (0.22 ± 0.16) mm at the inline direction respectively. Those values are lower than 1%/1 mm as reported by Nakamura et al.^5^ who showed that only small changes occurred due to the gimbal movement concerning the beam profiles. Their approach was based on two‐point comparisons to determine the value of FWHM, flatness, symmetry, and penumbra. A more detailed evaluation allowing a cause analysis of deviations is feasible by the approach chosen in the current study, which used other dosimetric parameters namely, OF, PDD, and 2D dose distributions to investigate the effect of pan and tilt on the dose distribution.

By utilizing Eqs. (3) and (4) to filter unwanted measurement data points during the PDD measurements, the mean detector position error could be constrained to 0.3 mm. The value is higher than the positioning accuracy of 0.1 mm stated in the phantom manual, since the manual does not state the multiple axes positioning accuracy. The determination of a threshold value needed by conducting multiple dry run tests. Based on the experience from the measurements we recommend performing dry run tests prior any complex measurements to avoid unnoticeable errors during the real measurements.

The gimbal swing created an extended SSD and a larger field size at the water surface as shown in Fig. 2. The SSD will extend by 0.9 mm and 1.8 mm at maximum pan *or* tilt and pan *and* tilt respectively. Such movements can create a systematic increase of dose uncertainties during treatment delivery. The value of the extended SSD is close to the AAPM TG 142^17^ error tolerance for the optical distance indicator (ODI) which is 2 mm. Several studies showed that a combination of extended SSD and larger field size would contribute to changes in OFs, PDD curves, and 2D dose distributions.^17^, ^18^

Relative OFs were calculated by dividing the detector reading of each field size measurement to that of the reference field size of 100 mm × 100 mm at each pan‐tilt measurements. Therefore, the OF deviation at 100 mm × 100 mm was always zero and the deviation decreases as the field sizes changed toward the reference field size of 100 mm × 100 mm. Due to the small deviation, the shape of the OF curves was also similar with the reference condition. Therefore, the scatter patterns that occurred during the measurements were similar with its reference condition.^18^ The tolerable deviation of OFs with the reference condition was 1%^17^ and the maximum deviation value of (−0.11 ± 0.33)% was still below the tolerance value. The measured dose during the OF measurement also showed a lower dose value, since the SSD was further than the reference condition. The differences were less than 0.5% except for 10 × 10 mm^2^ field size [Figs. 5(c) and 5(d)].

After filtering and correcting,^22^ the measured PDD data points using Eqs. (3) and (4) it was found that all PDD curves beyond the buildup region were higher than the references. The average deviations for all field sizes are 0.58 ± 0.26% and 0.54 ± 0.25% for the pan‐*or‐*tilt and pan‐*and‐*tilt position respectively. This result confirms that the gimbal movements affect the PDD curves. Figure 8 showed that combinations of the extended SSD and beam angle change caused this increase.^18^ The mean deviation of the PDD curve at pan *and* tilt is higher than pan *or* tilt, which shows that pan‐tilt movement has an effect on the PDD. An increase of the PDDs beyond the depth of maximum dose and a lower dose within the buildup region indicates that the contamination of charge particles and low energy photons during pan‐tilt was decreased^17^, ^18^, ^23^, ^24^, ^25^ compared to the reference condition. Similar situations also occur for the PDD distribution below the physical wedge,^25^ since it filters the contaminant scatter from the head and produces more penetrating radiation beams.

The 2D dose profile evaluation using the pixel‐to‐pixel approach showed that only 30% of the 320 fields had > 95% points that passed dose difference criteria 1%–4%. Figure 10 showed that the penumbra region was the main contributor to the failure. The pan‐tilt movements will influence the dose distribution in the penumbra region, which could create a dose difference up to 8%. Nevertheless, the mean dose deviations from the pixel‐to‐pixel comparison were less than 1%. Excluding the penumbra region and considering only 80% of the field size during evaluation, resulted in a similar mean dose deviation at a lower standard deviation. This concluded that the source of the high standard deviation was coming from the penumbra region. Table 2 shows that the mean deviation at pan *and* tilt is also higher than at pan *or* tilt position since the beam angle is higher at pan *and* tilt position.

The mean deviation of the 2D dose distribution, OF and PDD are higher at pan *and* tilt position than at pan *or* tilt position. This occurred due to the effective SSD and field size for pan *and* tilt being larger than at pan *or* tilt position. These results strengthen the fact that a small increase on SSD could give an effect on the dose distribution.

The measurements showed that the dose deviation at pan *and* tilt position are higher than at pan *or* tilt. This occurred due to longer SSD and a higher pan‐tit angle relative to the water surface. Another important aspect that should be considered during treatments that utilizing pan‐tilt tracking is the beam path [Figs. 2, 3 and Eq. (2)]. The shift for maximal pan/tilt of each point depended on its depth *d* according to Eq. (2) (Fig. 11). A lateral shift compared to the reference depth of 2.2 mm, 4.4 mm, and 8.7 mm at depth of *d *= 50 mm, 100 mm and 200 mm, respectively, were observed. Due to a changed radiation path based on the different beam angle, there might be a risk to irradiate sensitive organs proximal or distal to the isocenter. Dose deviations and beam path should be considered carefully while implementing tracking using pan‐tilt movements since this effect is not visible in the TPS. However, the dose distribution within the target is only influenced by the changes of the penumbra, which is less than 1 mm in term of distance deviation (Fig. 3). These results indicated that the dose distribution at the target edge region and the OAR beyond the target depth would be affected the most. Increasing the CTV‐PTV margin could increase dose coverage at the CAX plane during tracking but would be contra productive with the aim of tracking treatment purpose.

The effect of gimbal motion during tracking in the current study was performed at the maximum position and could be considered a worst‐case scenario. Therefore, further studies regarding the effect of gimbal motion in a real treatment setting are very important to assess the accuracy of the calculated and delivered dose distribution during the entire motion.

The pan‐tilt motion effect measured in this study is limited to the ideal conditions in a water phantom. The difference of each fundamental dosimetry parameter to the reference is less than 1%. The pan‐tilt motion effect measured in this study is limited to the ideal conditions in a water phantom. The difference of each fundamental dosimetry parameter to the reference is less than 1%. However, the conditions are much different in a real clinical condition due to the tumor motion direction and the shape of the patient which are not ideally represented by the phantom. The act of shifting the beam alone will not give the accurate dose calculation. The target motion during treatment will influence the dose coverage within the target and its surrounding OAR. For example, the target motion in parallel direction will create over‐ and under‐dose of the target due to the target being no longer at the SAD point.

The dose located in the target plane was not suffering a large dose difference at ring and gantry 0^o^ and the dose difference was less than 1%. However, the dose above and below the target shows more dose deviation due to the path difference, and the separation of the isocenter and the target dose (Fig. 2). The edge of the beam was suffering large deviation (Fig. 11) even though the beam shifting was applied. Taking the depth of the target as the reference CAX, the shift of the beam position above or below the target could be estimated using Eq. (2). The dose profile shifts at a dose plane that 10 cm away from the target is 1.7 mm for pan and tilt angle 1^o^. Considering the dose difference and the shift from this study, shifting the beam without taking into account the pan and tilt angle is relatively safe. However, the planner should consider the dose profile shift while adding the PTV margin of the target and OAR as a safety measure.

Preliminary studies regarding the implementation of the gimbal motion are currently ongoing. Additionally, the preliminary data show that the passing rates for pan *and* tilt angle 1^o^ dose calculation with and without considering the pan *and* tilt had a gamma passing rate > 99.2% using gamma criterion 3%/3 mm.^26^ Therefore, the expected 3D dose distribution difference at the gantry and ring 0^o^ is save for tracking angles less than 1^o^. Factors that contribute to the dose difference if the pan and tilt motion is not implemented are the position of the dose plane from the target, the ring and gantry angle, and the pan and tilt tracking directions and angle.

### 4.A Future study

More detailed works to simulate the real clinical situation are required to estimate a dose calculation closer to the real situation. New CT datasets and MU distributions corrected for the pan‐tilt position cannot be generated manually but require dynamic adjustment as part of the dose calculation. Nevertheless, this fundamental data can illustrate the effect of the pan‐tilt motion in the ideal situation and can be used as a precaution how to implement the pan‐tilt motion for the treatment in the absence of appropriate 4D TPS.

A feasibility study regarding the implementation of the pan and tilt motion in a TPS using image transformations of the CT data outside the TPS while implementing the ring and gantry rotations within the TPS was done.^26^ Full implementation of the approach in a TPS requires transformation of the CT dataset according to the pan and tilt orientation but does not need any modification of the TPS dose calculation algorithm, which makes the implementation is much easier.

## 5 CONCLUSIONS

The dose deviation for pan *and* tilt motion are higher than for gimbals moved in pan *or* tilt due to a longer SSD and a higher pan‐tit angle relative to the water surface. The impact of VERO gimbal movement on 2D dose profile, OF and PDD were less than 1%, 0.5%, and 0.5% respectively. The penumbra region is greatly influenced, at the investigated maximal gimbal motion with dose differences up to 8% against the reference position. There is also a shift of the radiation path that depends on the depth relative to the isocenter, which can influence OARs distal to the target volume. Considering the gimbal motion in the dose calculation would be beneficial to improve the accuracy of treatment delivery.

## ACKNOWLEDGMENTS

The presented work was performed by the first author HP in fulfillment of the requirements for obtaining the degree “Dr. rer. biol. hum.” at Friedrich‐Alexander‐Universität (FAU) in conjunction with the RISET‐PRO scholarship program of the Ministry of Research, Technology and Higher Education of the Republic Indonesia.

## CONFLICT OF INTEREST

The authors declare that there are no conflicts of interest in connection with this work.
